# CYP2C19 metabolic estrogen phenotypes and endometriosis risk in Brazilian women

**DOI:** 10.1016/j.clinsp.2023.100176

**Published:** 2023-03-07

**Authors:** Jamila Alessandra Perini, Daniel Escorsim Machado, Jéssica Vilarinho Cardoso, Vanessa Camara Fernandes, Claudio José Struchiner, Guilherme Suarez-Kurtz

**Affiliations:** a Laboratório de Pesquisa de Ciências Farmacêuticas (LAPESF), Universidade do Estado do Rio de Janeiro (UERJ zona oeste), Rio de Janeiro, RJ, Brazil; b Coordenação de Pesquisa, Instituto Nacional de Câncer, Rio de Janeiro, RJ, Brazil; c Escola de Matemática Aplicada, Fundação Getúlio Vargas, Rio de Janeiro, RJ, Brasil

Endometriosis is a chronic disease characterized by the presence of endometrium-like tissue outside the uterine cavity, associated with pelvic pain and infertility. The prevalence ranges between 2% and 10% in women of reproductive age and 30%–50% among infertile women [1]. The pathophysiology of endometriosis is still a matter of investigation, but it is clear that estrogen-mediated alterations play a major role as drivers of chronic inflammation, promoting endometriotic cell survival and lesion progression [1]. Candidate-gene and genome-wide studies identified a number of genetic factors affecting susceptibility to endometriosis, including Single Nucleotide Polymorphisms (SNPs) in *CYP2C19*, the gene encoding the cytochrome P450 2C19 (CYP2C19) protein. CYP2C19 is a major drug-metabolizing enzyme, also known to contribute to estrogen metabolism [2,3], such that functional *CYP2C19* polymorphisms that lead to changes in estrogen levels might affect susceptibility to endometriosis. Indeed, rs4244285, the SNP defining the no-function CYP2C19*2 star allele, has been reported to associate with increased endometriosis risk [4], [5], [6], [7], whereas rs12248560, which defines the increased function CYP2C19*17 has been associated with decreased risk of endometriosis development [5, 6]. These associations, however, have not been confirmed in other studies [7, 8]. Importantly, carriage of either CYP2C19*2 or CYP2C19*17 in homozygosis or heterozygosis predicts distinct CYP2C19 metabolic phenotypes, whereas compound heterozygosis for these SNPs associates with reduced CYP2C19 activity, despite the presence of the increased function CYP2C19*17 allele [9, 10]. The authors investigated the influence of genotype-inferred CYP2C19 metabolic phenotypes, rather than individual *CYP2C19* SNPs, on endometriosis risk in Brazilian women.

The study cohort comprised endometriosis patients (n = 180) and control women (n=176) with a negative diagnosis of endometriosis by laparoscopy or laparotomy. The women were recruited at two reference hospitals of the Brazilian Public Health System in Rio de Janeiro. Most participants had been enrolled in a previous report [7], which provides details of approval of the study protocol and written informed consent by both institutions´ review boards and the criteria adopted for diagnosis of endometriosis and Deeply Infiltrating Endometriosis (DIE). DIE was observed in 100 patients.

Participants were genotyped for rs4244285G>A (GRCh38.13 chr 10:94781859, CYP2C19*2) and rs12248560C>T (GRCh38.13 chr 10:94761900, CYP2C19*17) in *CYP2C19*, using TaqMan allele discrimination probes and a 7500 Real-Tyme System. CYP2C19 metabolic phenotypes were assigned according to the Clinical Pharmacogenetics Implementation Consortium guidelines [9], based on the CYP2C19 diplotypes comprising the *2, *17 and *1 (default) alleles, as follows: normal metabolizer (NM: diplotype *1/*1), intermediate metabolizer (IM: *1/*2 or *2/*17), poor metabolizer (PM: *2/*2), rapid metabolizer (RM: *1/*17) and ultrarapid metabolizer (UM: *17/*17). Pearson's Chi-Square test (χ^2^) was performed to assess the possible difference in *CYP2C19* frequency distribution. Odds Ratio (OR) with their 95% CI was used to evaluate the association of CYP2C19 phenotypes with endometriosis risk, either considering all cases or DIE. The Cochran-Armitage trend test was performed to assess the association between phenotypes and endometriosis risk.

Table 1 shows the distribution of *CYP2C19* alleles in the study cohorts. The Minor Allele Frequency (MAF) of rs4244285 (CYP2C19*2) and rs12248560 (CYP2C19*17) in the control group were similar to previous data for healthy Brazilians [11]. Considering the frequency distribution of the *CYP2C19* star alleles (*2, *17 and default *1), there was a significant difference between controls and overall cases (p=0.013) or DIE patients (p = 0.007). CYP2C19*2 had a higher frequency in all cases and DIE patients than controls, while the opposite was observed for CYP2C19*17. The distribution of diplotype-predicted CYP2C19 phenotypes (Table 1; Supplementary Fig. 1) differed nominally (p = 0.070) in controls *versus* overall endometriosis cases, but significantly between controls and DIE patients (p = 0.040). However, the Cochran-Armitage test revealed highly significant trends for an association of CYP2C19-predicted phenotypes with susceptibility to endometriosis, whether overall cases (p = 0.004) or DIE patients (p = 0.005). Thus, the susceptibility of disease increased progressively as the assigned CYP2C19 metabolic activity decreased from UM (6.8% controls, 2.8% all cases and 4.0% DIE) to PM (1.1% controls, 4.4% all cases and 7.0% DIE) phenotypes ( Table 1).

**Table 1:** Distribution of *CYP2C19* alleles, diplotypes and metabolic phenotypes.

	Controls (n = 176)	All cases (n = 180)	p-values^a^	DIE (n = 100)	p-values^a^
Star alleles	Allelic frequency (95% CI)			Allelic frequency (95% CI)	
*1 (default)	0.696 (0.646‒0.742)	0.694 (0.645‒0.740)		0.650 (0.582‒0.713)	
*2 (rs4244285G>A)	0.111 (0.082‒0.148)	0.172 (0.137‒0.215)	**0.013**	0.205 (0.155‒0.266)	**0.007**
*17 (rs12248560C>T)	0.193 (0.155‒0.238)	0.133 (0.102‒0.172)		0.145 (0.103‒0.200)	
Phenotypes (Diplotypes)	Phenotype frequency (95% CI)			Phenotype frequency (95% CI)	
NM (*1/*1)	0.506 (0.431‒0.581)	0.500 (0.425‒0.575)		0.440 (0.341‒0.543)	
IM (*1/*2 + *2/*17)	0.199 (0.143‒0.265)	0.256 (0.194‒0.326)		0.270 (0.186‒0.368)	
PM (*2/*2)	0.011 (0.001‒0.040)	0.044 (0.019‒0.085)	0.070	0.070 (0.029‒0.139)	**0.040**
RM (*1/*17)	0.216 (0.155‒0.283)	0.172 (0.120‒0.235)		0.180 (0.110‒0.269)	
UM (*17/*17)	0.068 (0.036‒0.116)	0.028 (0.009‒0.064)		0.040 (0.011‒0.099)	

a p-values from Pearson's Chi-Square test. DIE, Deep Infiltrating Endometriosis; CI, Confidence Interval; NM, Normal Metabolizer; IM, Intermediate Metabolizer; PM, Poor Metabolizer; RM, Rapid Metabolizer; UM, Ultrarapid Metabolizer.

Logistic regression (Table 2) confirmed the association of the PM phenotype with a higher risk of endometriosis, either considering all cases (OR = 4.0) or DIE patients (OR = 7.1 for DIE) compared to the NM phenotype. However, the Confidence Intervals (95% CI) for all cases included the null (OR = 1), while for DIE cases the CYP2C19 pathway may be key to the susceptibility of severe disease. The CYP2C19 is a target relevant because it participates in the estrogen conversion process to estradiol [2, 3], and endometriosis is an estrogen-dependent disease [12, 1]. No other significant associations were detected. The authors suggest that larger sample sizes are required to achieve the necessary power to fully describe the association of endometriosis risk with CYP2C19 metabolic activity.

**Table 2:** Regression analysis of association of CYP2C19 phenotypes with endometriosis risk.

CYP2C19 phenotypes^a^	OR (95% CI)^b^	
	Overall cases	DIE
PM	4.00 (0.82‒20.00)	**7.14 (1.41‒33.33)**
IM	1.30 (0.76‒2.22)	1.56 (0.84‒2.86)
RM	0.81 (0.46‒1.41)	0.96 (0.49‒1.85)
UM	0.41 (0.14‒1.22)	0.68 (0.21‒2.22)

a Assigned according to CYP2C19 diplotypes.

b OR, Odds Ratio; CI, Confidence Interval; NM, Phenotype used as reference; bold type indicates p<0.05. DIE, Deep Infiltrating Endometriosis; NM, Normal Metabolizer; IM, Intermediate Metabolizer; PM, Poor Metabolizer; RM, Rapid Metabolizer; UM, Ultrarapid Metabolizer.

Collectively, the present results are consistent with previous reports of increased susceptibility to endometriosis, both overall cases and DIE[4], [5], [6], [7], in carriers of the no-function rs4244285 SNP, and decreased risk in carriers of the increased function rs12248560 [4, 5]. Importantly, however, the observed trend for progressive decline in endometriosis susceptibility as predicted CYP2C19 activity increases is a novel finding, which points to distinct risk-altering effects associated with carriage of rs4244285 or rs12248560 in homozygosis, heterozygosis or compound heterozygosis. This is most evident in the considerably higher OR for endometriosis in rs4244285 homozygotes (PM phenotype) compared to rs4244285 heterozygotes or rs4244285/rs12248560 compound heterozygotes (both predicting the IM phenotype) [10]. Thus, the identification of different CYP2C19 phenotypes involved in the biological process of DIE may have implications in the diagnosis, identifying risk groups and therapeutic targets, given that, of the many biomarkers proposed in peripheral blood and endometrium, not one has been validated for endometriosis. [13] In addition, DIE can be considered a different phenotype of the same disease, shared with endometriomas and peritoneal lesions. [14] There is the heterogeneity of estrogen receptor α and progesterone receptor distribution in lesions of DIE in untreated women, or during exposure to various hormonal treatments [15] suggesting genetic variability in the prognosis and risk of endometriosis.

Finally, the present results support the notion that *CYP2C19* polymorphisms might exert their endometriosis risk-altering effects through influence on estrogen metabolism [[4], [5], [6], [7], 16], such that higher levels of estrogen in rs4244285 carriers, such as IMs and especially PMs, would increase susceptibility to endometriosis. Conversely, increased CYP2C19 activity in RMs and UMs, carriers of rs12248560, would reduce estrogen levels, and thereby diminish susceptibility to endometriosis.

## Declaration of Competing Interest

The authors declare no conflicts of interest.

## Appendix Supplementary materials

Image, application 1
