# Supplementary material for: CYP2C19 metabolic estrogen phenotypes and endometriosis risk in Brazilian women
Source: Clinics (Sao Paulo). 2023 Mar 7;78:100176. doi: 10.1016/j.clinsp.2023.100176 (PMC10018429; doi:10.1016/j.clinsp.2023.100176)
Supplement: Supplementary file 1 [file mmc1.docx]

CLINICS-D-22-00621_Supplementary Material

**Supplemenary Figure 1** Distribution of assigned CYP2C19 phenotypes in controls, overall endometriosis cases and DIE patients. NM, Normal Metabolizer; IM, Intermediate Metabolizer; PM, Poor Metabolizer; RM, Rapid Metabolizer; UM, Ultrarapid Metabolizer.

**
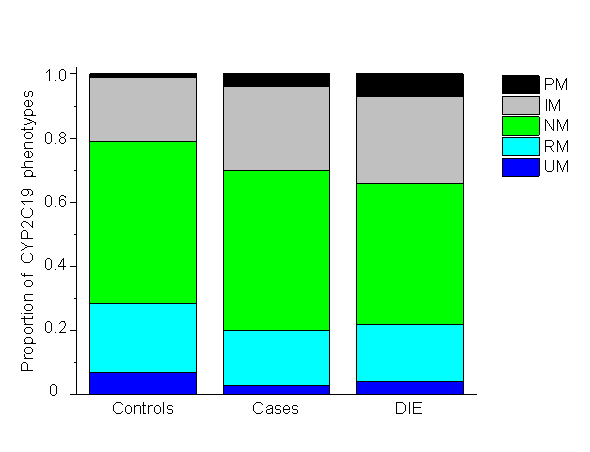
**
